# The effects of bicarbonated versus acetated Ringer's solutions on acid-base status and kidney injury following orthotopic liver transplantation: Protocol for a single-centre, randomised controlled trial (The BETTER trial)

**DOI:** 10.3389/fsurg.2022.1019570

**Published:** 2022-10-20

**Authors:** Cheng Lv, Bin Zhou, Donghua Zhang, Jiajia Lin, Lingling Sun, Zhenzhen Zhang, Yuan Ding, Rong Sun, Jie Zhang, Chuyao Zhou, Li Zhang, Xuan Wang, Lu Ke, Weiqin Li, Baiqiang Li

**Affiliations:** ^1^ Department of Critical Care Medicine, Jinling Hospital, Nanjing University School of Medicine, Nanjing, China; ^2^ Department of Anaesthesiology, Jinling Hospital, Nanjing University School of Medicine, Nanjing, China; ^3^ Department of Oncology Surgery, Jinling Hospital, Nanjing University School of Medicine, Nanjing, China; ^4^ Department of Laboratory Medicine, Jinling Hospital, Nanjing University School of Medicine, Nanjing, China

**Keywords:** bicarbonated Ringer's solution, liver transplantation, acid-base status, acute kidney injury, acetated Ringer's solution

## Abstract

**Background:**

The ideal crystalloid fluid of choice for fluid therapy during liver transplantation is unknown. Conventional balanced crystalloids are buffered with organic anions, which requires liver metabolism to prevent matabolic acidosis and protect renal function. Therefore they can not function properly during liver transplantation. On the contrary, the bicarbonated Ringer's solution (BRS) can maintain acid-base status regardless of liver function. In this study, we aimed to test the hypothesis that, in patients undergoing orthotopic liver transplantation, compared with acetated Ringer's solutions (ARS), perioperative fluid therapy with BRS could better maintain the acid-base status.

**Methods:**

This is a prospective, single-centre, randomised controlled trial. 72 eligible patients will be randomised to receive either BRS or ARS perioperatively. The primary endpoint is the difference in standard base excess (SBE) before and after operation. Secondary endpoints include the incidence of acute kidney injury (AKI) within 48 h post operation and free and alive days to day 14 for intensive care admission, invasive ventilation, vasopressors, and renal replacement therapy (RRT).

**Discussion:**

Metabolic acidosis is common perioperatively, potentially leading to decreased renal blood flow and reduced glomerular filtration rate. The use of balanced solutions can prevent hyperchloremic metabolic acidosis, thereby avoiding AKI in some patients. However, during liver transplantation, when well-functioning liver metabolism is lacking, the organic anions in conventional balanced solutions may remain strong anions and thus fail to maintain the acid-base status, but no solid clinical evidence exists now. This study will, for the first time, provide evidence on the relative effects of BRS vs. ARS on acid-base status and renal injury in patients undergoing liver transplantation.

**Clinical Trial Registration:**

The trial has been registered at the Chinese Clinical Trials Registry (ChiCTR2100046889) on 29 May 2021.

## 1. Background

Acute kidney injury (AKI) is a common complication following liver transplantation, occurring in 29%–60% of pateints (1–4). Once it develops, regardless of the severity of AKI, it is significantly associated with increased mortality (5). A long-term cohort study conducted by the National Institute of Diabetes, Digestive and Kidney Diseases showed that, compared with patients with normal renal function, those with AKI had a hazard ratio of 1-year mortality up to 3.59 (6). The development of AKI is due to multiple factors (7–14), one of which is intraoperative and postoperative infusion of supraphysiologic chlorinated liquids (15).

During liver transplantation, fluid therapy, including crystalloids and colloids, is considered the cornerstone of perioperative management to maintain normal blood volume and preserve renal perfusion (15). The RELIEF study shows that patients undergoing liver transplantation and other major abdominal surgeries had an average fluid infusion of 3,500 ml intraoperatively and 6,146 ml within 24 h after the operation, respectively, and most of the intravenous fluids are crystalloid (16, 17).

However, the ideal crystalloid during liver transplantation remains uncertain (18). By now, normal saline (NS, 0.9% sodium chloride) is most frequently used (19, 20). Nevertheless, the chloride concentration of NS (154 mmol/L) is significantly higher than that of plasma (94–111 mmol/L), which may lower plasma strong ion difference (SID), leading to metabolic acidosis and impaired renal perfusion (21, 22). A possible alternative is balanced solution (chloride limited crystalloid) (23), which, as shown in the SMART trial, could reduce adverse kidney events in critically ill patients when compared with NS (24).

In practice, the most commonly used balanced crystalloids are buffered with organic anions, such as lactate, acetate, or malate, all of which require liver metabolism to increase SID (25). During liver transplantation, liver metabolism is suspended during the anhepatic phase and severely compromised during the early neohepatic phase (26), thus organic anions buffered solution can not function properly, thereby inducing metabolic acidosis.

On the contrary, bicarbonated Ringer's solution (BRS), introduced 11 years ago, directly replaces chloride with bicarbonate. It can increase SID regardless of liver metabolism. Previous pharmacokinetic studies showed that BRS could correct metabolic acidosis faster than lactated or acetated Ringer's solution (ARS) in shock models (26). Taken together, compared with conventional organic anion buffered balanced solutions, BRS may better maintain acid-base status during liver transplantation, but no evidence exists in the literature.

In this study, we aim to compare the effect of BRS and ARS on acid-base status and renal function in patients undergoing liver transplantation. The results of this study could also provide data to power a confirmatory Phase III study in the future.

## 2. Methods

### 2.1. Aim and objectives

The primary objective of this trial is to determine whether, compared to ARS, BRS can better maintain acid-based status as reflected by the change in standard base excess (SBE) levels in patients undergoing orthotopic liver transplantation.

Secondary objectives are to assess the impact of BRS on renal function and other clinical outcomes like the number of days alive and free of intensive care admission, invasive ventilation, vasopressors, and renal replacement therapy (RRT) to day 14 after operation. Laboratory results like bicarbonate, creatinine and neutrophil gelatinase-associated lipocalin (NGAL) levels will also be compared.

### 2.2. Study design

The present study is an investigator-initiated, single-centre, superiority, randomised controlled trial. This trial was registered at the Chinese Clinical Trials Registry (ChiCTR2100046889).

### 2.3. Trial committees

A Trial Management Committee (TMC) was formed, comprising the principal investigator and all the other co-investigators (clinical and non-clinical). The TMC is responsible for the day-to-day running of the trial. A writing and publication committee was organised for drafting the manuscript and submission of the manuscript to proper academic journals. It will also decide on the authorship of this study. An independent data safety and monitoring board (DSMB) consisting of a surgeon, an anesthesiologist, and a statistician will be organised to oversee all subjects' safety. The DSMB will review the safety report regularly and have the right to stop the trial early because of concerns about participant safety. DSMB members will not be involved in the study conduct, and all the processes will be independent of the investigators.

### 2.4. Study population

All adult patients undergoing orthotopic liver transplantation surgery admitted to the Jinling Hospital, Nanjing University, will be assessed for eligibility after admission. The inclusion and exclusion criteria are as follows:

#### 2.4.1. Inclusion criteria

1. Recipients undergoing orthotopic liver transplantation surgery;
2. Age ≥18 years.
3. Written informed consent.

#### 2.4.2. Exclusion criteria

1. Combined transplantation;
2. Patients receiving RRT within 1 week before operation;
3. Patients with increased serum creatinine (SCr) levels within 1 week before operation (Defined as SCr increased by more than 1.5 times or 26.5 μmol/L (0.3 mg/dL) from baseline. Baseline was defined as SCr levels obtained within 6 months, if not available, the upper limit of normal was set for baseline at 90 μmol/L for females/110 μmol/L for males);

A patient will be considered eligible if he/she meets the inclusion criteria and does not meet any of the exclusion criteria.

### 2.5. Randomisation

The randomisation sequence will be generated by an independent statistician using SAS Version 9.4 with a fixed block size (block size = 4). Randomisation will be stratified by the surgical methods adopted (classical orthotopic liver transplantation vs. piggyback orthotopic liver transplantation). Allocation will be in a 1:1 ratio.

### 2.6. Blinding method

Due to the specific double-packaging of bicarbonated Ringer's solution for stabilising the concentration of bicarbonate radical, blinding to investigators will not be applicable. However, research personnel, ICU staff and other caregivers will not have access to the randomisation schedule. Allocation concealment will be maintained by using a secure, password-protected study website to randomise consenting patients.

### 2.7. Study interventions

The only difference between the two study groups exists in the choice of perioperative intravenous isotonic crystalloid: bicarbonated Ringer's solution (BRS group) vs. acetated Ringer's solution (ARS group). The compositions of each crystalloid solution are displayed in Supplementary Table S1.

• *Group 1: BRS group*
  Patients will receive bicarbonate Ringer's solution for fluid therapy during and within 48 h post operation.
• *Group 2: ARS group*
  Patients will receive acetate Ringer's solution for fluid therapy during and within 48 h post operation.

### 2.8. Management of liver transplantation

First, both groups received general standard of care involving the following key components (27). The operating room temperature will be set at 22°C and standard warming measures be used for all patients. All patients will receive standard general anaesthesia induced by midazolam, sufentanil, etomidate, cisatrcurium and maintained by propofol, remifentanil, sevoflurane and additional injection of sufentanil. An arterial line and a central venous catheter will be inserted before induction of anaesthesia. Mechanical ventilation will be performed routinely. During the perioperative period, all patients will receive intravenous fluids according to a standardised protocol, which includes basiliximab, antibiotics, esomeprazole and methylprednisolone. Transfusions will be administered at the discretion of the attending anaesthetist according to the National Blood Transfusion guidelines.

Throughout the operation, arterial blood gas will be measured every 60 min during the dissection phase, every 30 min during the anhepatic phase (including a 5-min pre-reperfusion blood gas) and every 30 min during the neohepatic phase for 1 h (including a 5-min post-reperfusion blood gas), then hourly until surgical closure. When pH is less than 7.2 or SBE is less than −10 mmol/L, additional 5% sodium bicarbonate will be infused with a total amount of 5% NaHCO_3_(ml) = (−2.3-SBE)*weight (kg)*0.42 to correct acidosis. The infusion will be started at a rate of 90 ml/h and discontinued with a targeted pH level of 7.35.

When the operation is completed, all the patients will be transferred to the surgical Intensive Care Units for postsurgical care. Both groups will receive standard treatment according to the guidelines after admission (27), including continuous vital signs recording, adequate fluid therapy, routine medical treatment (blood glucose control, antibiotics if needed and sedatives if required), organ support measures, etc. Organ failure would be assessed on a daily basis according to the SOFA score.

Initiation of RRT should be based on the criteria described by Bellomo et al. (28). Patients who have AKI (at least 1.5 times increase in creatinine from known baseline value) and meet predefined specific criteria will be eligible for initiation of RRT.

### 2.9. Endpoints

#### 2.9.1. Primary outcome measure

The primary outcome measure is the change of SBE during the operation, reflected by the difference in SBE measure before and after the operation (ΔSBE = SBE_post_-SBE_pre_). SBE_pre_ will be measured before introduction of anaesthesia, and SBEpost will be measured shortly before transportation to the surgical ICU.

#### 2.9.2. Secondary outcome measures

##### 2.9.2.1. Part I: Process measures

1. The amount of BRS/ARS used during operation and within the first 2 days post operation;
2. Additional sodium bicarbonate used during operation and within the first 2 days post operation;
3. Serum acetic acid concentration before operation, 5 min post reperfusion of the donor liver, 0, 6, 12, 24 and 48 h post operation.

##### 2.9.2.2. Part II: Renal function-related measures

All the **baselines** mentioned below refer to data obtained within 24 h before operation.

1. Incidence of AKI within 48 h after operation. AKI is defined as current serum creatinine (SCr) increased 1.5 times baseline value OR with an increase greater than 26.5 µmol/L.
2. Serum Scr, Cys C levels at 0, 24 and 48 h post operation;
3. Serum NGAL levels at 0, 12, 24 and 48 h post operation;
4. Arterial pH, SBE, HCO_3_^−^, Lac, Cl^−^ at 5 min post reperfusion of the donor liver, 0, 6, 12, 24 and 48 h post operation;
5. Effective SID (eSID = [Na^+^] + [K^+^] + [Ca^2+^] + [Mg^2+^]-[Cl^−^]-[Lac^−^]) at 5 min post reperfusion of the donor liver, 0, 6, 12, 24 and 48 h post operation;
6. Requirement of new RRT within 14 days post operation;
7. RRT free and alive days to 14 days post operation.

##### 2.9.2.3. Part III: Clinical outcome measures

1. New receipt of organ support within 14 days post operation (new receipt of RRT; new receipt of mechanical ventilation (non-invasive included); new receipt of vasoactive agents);
2. New-onset organ failure within 14 days post operation;
3. ICU free and alive days within 14 days post operation;
4. Mortality to 14 days post operation.

### 2.10. Monitored parameters and data collection

**Figure 1:**
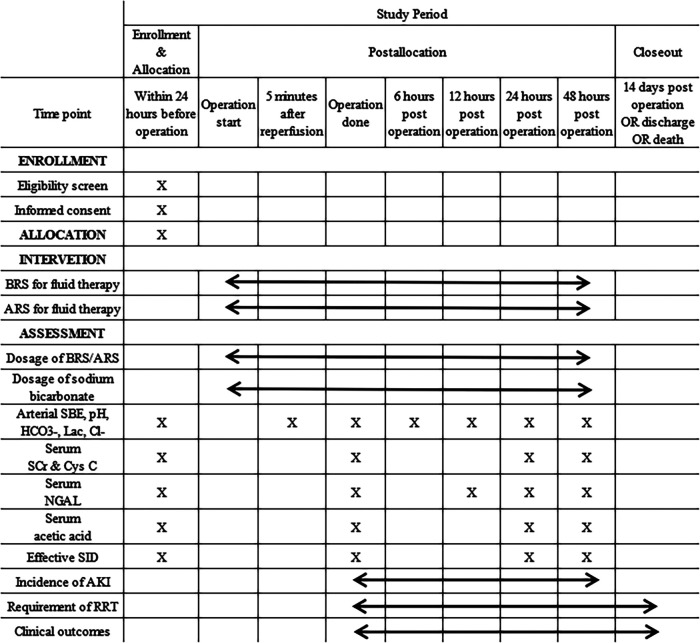
Schedule of enrolment, interventions, and assessments. BRS, bicarbonated Ringer's solution; ARS, acetated Ringer's solution; SBE, standard base excess; Lac, lactate; SCr, serum creatinine; Cys C, cystatin C; NGAL, neutrophil gelatinase-associated lipocalin; SID, strong ion difference; AKI, acute kidney injury; RRT, renal replacement therapy. *This figure is created by CL, JL and LK.

A web-based electronic database (Unimed Scientific, Wuxi, China) is used for data collection and storage. All data are input by the designated coordinator. Training for data entry was arranged by the provider of the electronic database before study commencement. The data required to be collected during different phases are shown in Figure 1.

### 2.11. Data management, sample size and statistical analysis

The principal investigator will be responsible for data management, safety, privacy, and quality. The reporting and presentation of this trial will follow the CONSORT guidelines (29). Based on the principle of intention to treat (ITT), a full-analysis set (FAS) will be performed on the population with outcome reporting. FAS will be used for the analysis of baseline characteristics and main therapeutic interventions. The safety set (SS) will include all enrolled patients to assess the safety profile of the study intervention.

According to the previous study (30) and our unpublished data, the change of SBE during operation is approximately −2 mmol/L with a standard deviation of 2.5 mmol/L in patients receiving ARS. We estimated that a sample size of 72 participants (36 per group) could provide 80% power at a two-sided alpha level of 0.05 to detect ≥1.7 mmol/L increase in the primary endpoint results from the use of BRS with a potential loss of 5% recruitments.

Descriptive statistics will be used to assess any marked baseline differences in demographics or outcome measures between the two groups. Comparisons of binary outcomes will be expressed as relative risk with 95% confidence intervals and comparisons of continuous outcomes as mean differences together with 95% confidence intervals. Comparisons will be made using *t*-test and ANOVA for repeated-measures or Wilcoxon rank-signed test and Kruskall-Wallis according to the underlying distribution for continuous data and Chi-square or Fisher's exact *T* test for categorical data, as appropriate. Two-sided 5% significance levels will be used to identify statistically significant results. All confidence intervals reported will be 95% confidence intervals.

### 2.12. Adverse events

All adverse events (AE) are required to be recorded and the DSMB will review all the safety profiles regularly during the study period. AEs will be reported in a uniform format through the electronic data capture system. The detection and report of the AEs will depend on the physicians involved in this trial.

### 2.13. Patient and public involvement

Patients or the public were not involved in the design, conduct, reporting, or dissemination plans of our research.

## 3. Discussion

Metabolic acidosis is common perioperatively, potentially leading to decreased renal blood flow and reduced glomerular filtration rate (31). The use of balanced solutions can prevent hyperchloremic metabolic acidosis, thereby avoiding AKI in some patients. However, in patients suffering hemorrhagic shock or undergoing major surgery, where the liver metabolism is impaired, the organic anions in conventional balanced solutions might remain strong anions and thus fail to maintain acid-base status. BRS, a novel balanced crystalloid buffered with bicarbonate rather than organic anions, may perform better in maintaining acid-base status under such circumstances. Paul et al. demonstrated that compared with ARS, BRS significantly reduced large acetate surges in cardiac surgical patients (32). Animal experiments and small clinical studies also observed similar phenomenons (33–35), but the impact on renal function was not assessed.

In patients undergoing liver transplantation, liver metabolism, essential for all organic anion buffered balanced solutions to function properly, is suspended in the anhepatic phase and severely compromised during the early neohepatic phase. BRS, therefore, is likely to benefit these patients by better sustaining the acid-base status and thereby protecting their renal function. However, no clinical study has investigated that yet. This study will, for the first time, provide evidence on the relative effects of BRS vs. ARS on acid-base state and renal injury in patients undergoing liver transplantation.

As the infusion of sodium bicarbonate to correct metabolic acidosis could significantly increase the pH and SBE, we worked out a standardised protocol for the use of sodium bicarbonate. The amount of sodium bicarbonate used perioperatively would also be recorded. Moreover, it is known that, compared with the piggyback technique, the classical surgical procedure involves excision of the recipient inferior vena cava (IVC), which requires cross-clamping of the IVC above and below the liver, as well as the portal vein. This results in a marked reduction in venous return and consequent haemodynamic instability (36). Therefore, patients undergoing classical liver transplantations would inevitably suffer more severe metabolic acidosis. However, both surgical approaches are widely used in the study site at the discretion of the surgical teams. Therefore, we adopted stratified randomisation to compensate for the confounding effect caused by surgical methods.

This trial has several strengths and limitations. This is the first randomised controlled trial providing high-level evidence evaluating the effect of bicarbonated Ringer's solution in patients undergoing liver transplantation. And the data will be reviewed by an independent data safety monitoring board to ensure the participants' safety. However, As a phase II RCT, the study has a relatively small sample size recruited from one centre and not sufficiently powered to detect a difffference in patient-centered outcomes like incidence of AKI post operation.

The present trial is sponsored by Jinling Hospital of Nanjing University, which is the municipal centre for liver transplantation performing more than 120 liver transplantations annually. Thus the study is expected to be concluded within a year.

## Trial status

The BETTER trial (protocol version 1.0, February 2021) will be conducted between 1 June 2021 and 31 December 2022.
