# Supplementary material for: The effects of bicarbonated versus acetated Ringer's solutions on acid-base status and kidney injury following orthotopic liver transplantation: Protocol for a single-centre, randomised controlled trial (The BETTER trial)
Source: Front Surg. 2022 Oct 20;9:1019570. doi: 10.3389/fsurg.2022.1019570 (PMC9630575; doi:10.3389/fsurg.2022.1019570)
Supplement: Supplementary file 1 [file Table1.docx]

**Supplemental Material**

Table S1: The compositions of bicarbonated Ringer’s solution and acetated Ringer’s solution

|  | Bicarbonated Ringer’s solution | Acetated Ringer’s solution |
| --- | --- | --- |
| Volume | 500ml | 500ml |
| NaCl | 2.92g | 3.00g |
| KCl | 0.15g | 0.15g |
| CaCl_2_·2H_2_O | 0.11g | 0.10g |
| MgCl_2_·6H_2_O | 0.10g | / |
| NaHCO_3_ | 1.175g | / |
| Sodium citrate·2H_2_O | 0.10g | / |
| Citric acid·H_2_O | About 0.07g | / |
| Sodium acetate | / | 1.90g |

*This table is created by CL, JL and LK.
